# 4-[(*E*)-4-Methoxy­benzyl­ideneamino]-3-{1-[4-(2-methyl­prop­yl)phen­yl]eth­yl}-1*H*-1,2,4-triazole-5(4*H*)-thione

**DOI:** 10.1107/S1600536809014949

**Published:** 2009-04-30

**Authors:** Hoong-Kun Fun, Samuel Robinson Jebas, K. V Sujith, Balakrishna Kalluraya

**Affiliations:** a X-ray Crystallography Unit, School of Physics, Universiti Sains Malaysia, 11800 USM, Penang, Malaysia; b Department of Studies in Chemistry, Mangalore University, Mangalagangotri, Mangalore 574 199, India

## Abstract

In the title compound, C_22_H_26_N_4_OS, the benzene rings of the (2-methyl­prop­yl)phenyl and 4-methoxy­phenyl units form dihedral angles of 66.85 (3) and 25.96 (3)°, respectively, with the triazole ring. The dihedral angle between the two benzene rings is 87.42 (2)°. The –CH(CH_3_) linkage is disordered over two orientations with occupancies of 0.907 (3) and 0.093 (3). An intra­molecular C—H⋯S hydrogen bond generates an *S*(6) ring motif. Inter­molecular N—H⋯S hydrogen bonds and C—H⋯π inter­actions are observed.

## Related literature

For the pharmaceutical applications of triazole compounds, see: Amir & Kumar (2007 ▶); Clemons *et al.* (2004 ▶); Demirbas & Ugurluoglu (2004 ▶); Demirbas *et al.* (2002 ▶); Johnston (2002 ▶); Shujuan *et al.* (2004 ▶). For bond-length data, see: Allen *et al.* (1987 ▶). For hydrogen-bond motifs, see: Bernstein *et al.* (1995 ▶). For the stability of the temperature controller used for the data collection, see: Cosier & Glazer (1986 ▶).
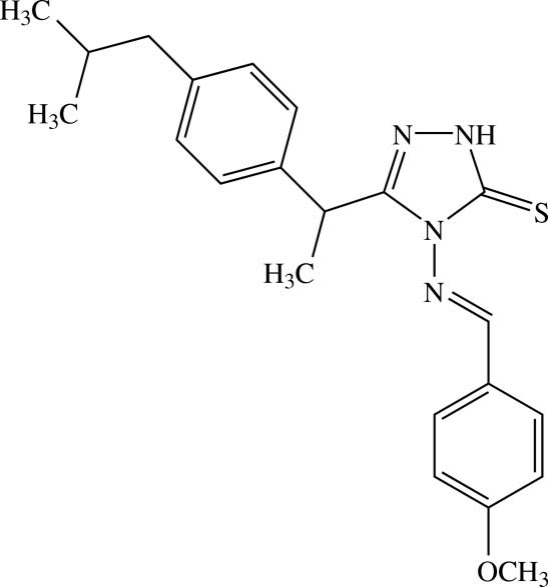


## Experimental

### 

#### Crystal data

C_22_H_26_N_4_OS
*M* *_r_* = 394.53
Triclinic, 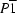

*a* = 7.9446 (1) Å
*b* = 11.1392 (2) Å
*c* = 12.3797 (2) Å
α = 77.769 (1)°
β = 79.025 (1)°
γ = 80.063 (1)°
*V* = 1041.08 (3) Å^3^
*Z* = 2
Mo *K*α radiation
μ = 0.18 mm^−1^
*T* = 100 K
0.50 × 0.27 × 0.13 mm

#### Data collection

Bruker SMART APEXII CCD area-detector diffractometer
Absorption correction: multi-scan (*SADABS*; Bruker, 2005 ▶) *T*_min_ = 0.918, *T*_max_ = 0.977
38166 measured reflections
9105 independent reflections
7573 reflections with *I* > 2σ(*I*)
*R*_int_ = 0.029

#### Refinement

*R*[*F* ^2^ > 2σ(*F* ^2^)] = 0.040
*wR*(*F* ^2^) = 0.111
*S* = 1.03
9105 reflections
281 parameters
H atoms treated by a mixture of independent and constrained refinement
Δρ_max_ = 0.65 e Å^−3^
Δρ_min_ = −0.29 e Å^−3^

### 

Data collection: *APEX2* (Bruker, 2005 ▶); cell refinement: *SAINT* (Bruker, 2005 ▶); data reduction: *SAINT*; program(s) used to solve structure: *SHELXTL* (Sheldrick, 2008 ▶); program(s) used to refine structure: *SHELXTL*; molecular graphics: *SHELXTL*; software used to prepare material for publication: *SHELXTL* and *PLATON* (Spek, 2009 ▶).

## Supplementary Material

Crystal structure: contains datablocks global, I. DOI: 10.1107/S1600536809014949/ci2785sup1.cif

Structure factors: contains datablocks I. DOI: 10.1107/S1600536809014949/ci2785Isup2.hkl

Additional supplementary materials:  crystallographic information; 3D view; checkCIF report

## Figures and Tables

**Table 1:** Hydrogen-bond geometry (Å, °)

*D*—H⋯*A*	*D*—H	H⋯*A*	*D*⋯*A*	*D*—H⋯*A*
N1—H1*N*1⋯S1^i^	0.87 (2)	2.39 (2)	3.2482 (8)	168 (1)
C15—H15*A*⋯S1	0.93	2.66	3.1947 (9)	117
C13—H13*A*⋯*Cg*1^ii^	0.96	2.77	3.5416 (12)	138
C12—H12*A*⋯*Cg*2^ii^	0.96	2.73	3.5417 (11)	142
C18—H18*A*⋯*Cg*2^iii^	0.93	2.80	3.5903 (10)	144
C22—H22*C*⋯*Cg*3^iv^	0.96	2.78	3.5783 (10)	142
C14*A*—H14*D*⋯*Cg*3^v^	0.96	2.88	3.769 (13)	155

Symmetry codes: (i) 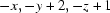; (ii) 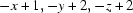; (iii) 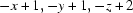; (iv) 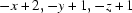; (v) 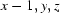. *Cg*1 is the centroid of the N1/N2/C2/N3/C1 ring, *Cg*2 is the centroid of the C4–C9 ring and *Cg*3 is the centroid of the C16–C21 ring.

**Fig. 1.**
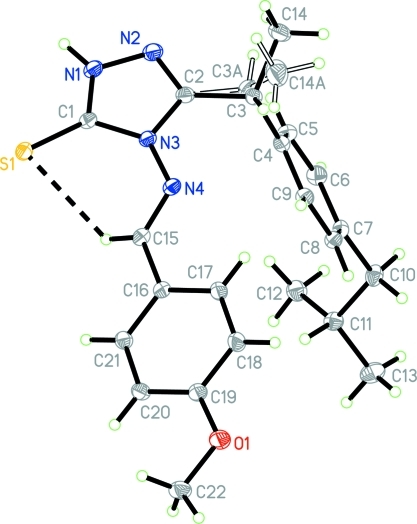
The molecular structure of the title compound, showing 50% probability displacement ellipsoids and the atom-numbering scheme. Both disorder components are shown. The dashed line indicates a hydrogen bond.

**Fig. 2.**
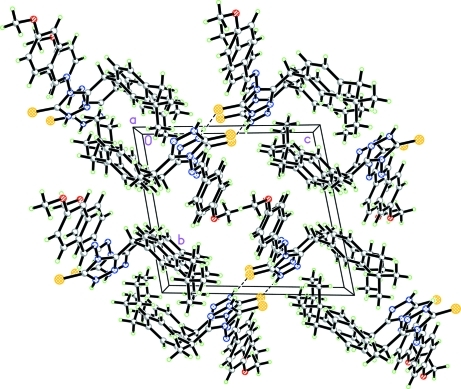
Part of the crystal packing of the title compound, viewed along the a axis. Hydrogen bonds are shown as dashed lines. Only the major disorder component is shown.

## supplementary crystallographic information

### Comment

Several compounds containing 1,2,4-triazole rings are well known as drugs. For example, fluconazole is used as an antimicrobial drug (Shujuan *et al.*, 2004), while vorozole, letrozole and anastrozole are non-steroidal drugs used for the treatment of cancer (Clemons *et al.*, 2004) and loreclezole is used as an anticonvulsant (Johnston 2002) agent. 1,2,4-Triazoles and their derivatives represent an overwhelming and rapidly developing field in modern heterocyclic chemistry. Similarly, ibuprofen belongs to the class of Non-Steroidal Anti-Inflammatory Drugs (NSAIDs) with antipyretic, anti-inflammatory and analgesic properties (Amir & Kumar, 2007). Schiff base derivatives of acetic acid hydrazides containing 1,2,4-triazol-5-one ring have displayed antitumor activity against breast cancer, while 2-phenyl ethylidenamino and 2-phenyl ethylamino derivatives of 4-amino-1,2,4-triazol-5-ones have been found to be effective towards non-small cell lung cancer, CNC and breast cancer (Demirbas *et al.*, 2002, 2004). Due to the progress that occurs in dealing with the chemistry of substituted 4-amino-1,2,4-triazole-3-thiones and their derivatives as well as their biological activities, we synthesized the title compound and herein report its crystal structure.

Bond lengths in the title molecule (Fig. 1) are found to have normal values (Allen *et al.*, 1987). The triazole ring is planar to within ±0.024 (1) Å. The dihedral angle formed by the triazole (N1/N2/C2/N3/C1) ring with the C4-C9 and C16-C21 benzene rings are 66.85 (3)° and 25.96 (3)°, respectively. The dihedral angle between the two benzene rings of 87.42 (2)°, indicates that these rings are oriented almost perpendicular to each other. An intramolecular C—H···S hydrogen bond generates an S(6) ring motif (Bernstein *et al.*, 1995).

The crystal packing is stabilized by intermolecular N—H···S hydrogen bonding together with weak C—H···π interactions (Table 1) (Fig 2).

### Experimental

The title compound, a Schiff base, was obtained by refluxing a mixture of 4-amino-5-[1-(4-isobutylphenyl)ethyl]-4*H*-1,2,4-triazole-3-thiol (0.01 mol), 4-methylbenzaldehyde (0.01 mol) in ethanol (50 ml) and 3 drops of concentrated Sulfuric acid for 3 h. The solid product obtained was collected by filtration, washed with ethanol and dried. It was then recrystallized using ethanol. Single crystals suitable for X-ray analysis were obtained by slow evaporation of an ethanol-*N*,*N*-dimethyl formamide (DMF) (3:1) solution.

### Refinement

The CH(CH_3_) unit is disordered over two orientations with occupancies of 0.907 (3) and 0.093 (3). N-bound H atoms were located in a difference Fourier map and were refined freely. C-bound H atoms were positioned geometrically [C-H = 0.93–0.97 Å] and refined using a riding model, with *U*_iso_(H) = 1.2*U*_eq_(C) and 1.5*U*_eq_(methyl C). A rotating-group model was used for methyl groups.

### Crystal data

**Table:**

C_22_H_26_N_4_OS	*Z* = 2
*M**_r_* = 394.53	*F*(000) = 420
Triclinic, *P*1	*D*_x_ = 1.259 Mg m^−^^3^
Hall symbol: -P 1	Mo *K*α radiation, λ = 0.71073 Å
*a* = 7.9446 (1) Å	Cell parameters from 9938 reflections
*b* = 11.1392 (2) Å	θ = 2.8–36.8°
*c* = 12.3797 (2) Å	µ = 0.18 mm^−^^1^
α = 77.769 (1)°	*T* = 100 K
β = 79.025 (1)°	Block, colourless
γ = 80.063 (1)°	0.50 × 0.27 × 0.13 mm
*V* = 1041.08 (3) Å^3^	

### Data collection

**Table:**

Bruker SMART APEXII CCD area-detector diffractometer	9105 independent reflections
Radiation source: fine-focus sealed tube	7573 reflections with *I* > 2σ(*I*)
graphite	*R*_int_ = 0.029
φ and ω scans	θ_max_ = 35.0°, θ_min_ = 1.9°
Absorption correction: multi-scan (*SADABS*; Bruker, 2005)	*h* = −12→12
*T*_min_ = 0.918, *T*_max_ = 0.977	*k* = −17→17
38166 measured reflections	*l* = −19→19

### Refinement

**Table:**

Refinement on *F*^2^	Primary atom site location: structure-invariant direct methods
Least-squares matrix: full	Secondary atom site location: difference Fourier map
*R*[*F*^2^ > 2σ(*F*^2^)] = 0.040	Hydrogen site location: inferred from neighbouring sites
*wR*(*F*^2^) = 0.111	H atoms treated by a mixture of independent and constrained refinement
*S* = 1.03	*w* = 1/[σ^2^(*F*_o_^2^) + (0.0555*P*)^2^ + 0.2526*P*] where *P* = (*F*_o_^2^ + 2*F*_c_^2^)/3
9105 reflections	(Δ/σ)_max_ = 0.001
281 parameters	Δρ_max_ = 0.65 e Å^−^^3^
0 restraints	Δρ_min_ = −0.29 e Å^−^^3^

### Special details

**Table:**

Experimental. The crystal was placed in the cold stream of an Oxford Cyrosystems Cobra open-flow nitrogen cryostat (Cosier & Glazer, 1986) operating at 100.0 (1) K.
Geometry. All e.s.d.'s (except the e.s.d. in the dihedral angle between two l.s. planes) are estimated using the full covariance matrix. The cell e.s.d.'s are taken into account individually in the estimation of e.s.d.'s in distances, angles and torsion angles; correlations between e.s.d.'s in cell parameters are only used when they are defined by crystal symmetry. An approximate (isotropic) treatment of cell e.s.d.'s is used for estimating e.s.d.'s involving l.s. planes.
Refinement. Refinement of *F*^2^ against ALL reflections. The weighted *R*-factor *wR* and goodness of fit *S* are based on *F*^2^, conventional *R*-factors *R* are based on *F*, with *F* set to zero for negative *F*^2^. The threshold expression of *F*^2^ > σ(*F*^2^) is used only for calculating *R*-factors(gt) *etc*. and is not relevant to the choice of reflections for refinement. *R*-factors based on *F*^2^ are statistically about twice as large as those based on *F*, and *R*- factors based on ALL data will be even larger.

### Fractional atomic coordinates and isotropic or equivalent isotropic displacement parameters (Å^2^)

**Table:**

	*x*	*y*	*z*	*U*_iso_*/*U*_eq_	Occ. (<1)
S1	0.28546 (3)	0.91565 (2)	0.481988 (17)	0.01833 (5)	
O1	1.15746 (9)	0.44217 (7)	0.65279 (6)	0.02374 (14)	
N1	0.01719 (10)	0.92699 (7)	0.65460 (6)	0.01744 (13)	
N2	−0.04031 (10)	0.86893 (8)	0.76193 (6)	0.01929 (14)	
N3	0.23009 (9)	0.79552 (7)	0.70327 (6)	0.01442 (12)	
N4	0.38158 (9)	0.71119 (7)	0.70940 (6)	0.01542 (12)	
C1	0.17881 (10)	0.88191 (8)	0.61363 (7)	0.01473 (13)	
C2	0.09098 (11)	0.78783 (8)	0.78932 (7)	0.01672 (14)	
C3	0.09472 (18)	0.69214 (14)	0.89541 (11)	0.0151 (2)	0.907 (3)
H3A	0.1528	0.6133	0.8746	0.018*	0.907 (3)
C14	−0.09117 (14)	0.67381 (11)	0.95160 (9)	0.0238 (3)	0.907 (3)
H14A	−0.1459	0.6413	0.9034	0.036*	0.907 (3)
H14B	−0.1551	0.7520	0.9651	0.036*	0.907 (3)
H14C	−0.0886	0.6167	1.0214	0.036*	0.907 (3)
C3A	0.061 (2)	0.7326 (14)	0.9050 (13)	0.021 (2)	0.093 (3)
H3AA	−0.0491	0.7717	0.9410	0.025*	0.093 (3)
C14A	0.0488 (19)	0.6028 (14)	0.9039 (10)	0.037 (4)	0.093 (3)
H14D	−0.0359	0.6001	0.8588	0.056*	0.093 (3)
H14E	0.0149	0.5605	0.9790	0.056*	0.093 (3)
H14F	0.1593	0.5630	0.8731	0.056*	0.093 (3)
C13	0.73078 (13)	0.89352 (10)	1.25067 (9)	0.02513 (18)	
H13A	0.8040	0.9571	1.2222	0.038*	
H13B	0.8007	0.8137	1.2582	0.038*	
H13C	0.6668	0.9055	1.3225	0.038*	
C4	0.19848 (11)	0.72643 (8)	0.97397 (7)	0.01640 (14)	
C5	0.13401 (11)	0.81940 (9)	1.03721 (7)	0.01883 (15)	
H5A	0.0272	0.8665	1.0283	0.023*	
C6	0.22794 (12)	0.84237 (9)	1.11351 (7)	0.01903 (15)	
H6A	0.1821	0.9037	1.1556	0.023*	
C7	0.39036 (11)	0.77416 (8)	1.12736 (7)	0.01561 (14)	
C8	0.45494 (11)	0.68414 (8)	1.06158 (7)	0.01569 (14)	
H8A	0.5638	0.6392	1.0679	0.019*	
C9	0.36033 (11)	0.65989 (8)	0.98659 (7)	0.01643 (14)	
H9A	0.4061	0.5984	0.9445	0.020*	
C10	0.49455 (13)	0.79371 (8)	1.21069 (7)	0.01946 (16)	
H10A	0.5707	0.7175	1.2312	0.023*	
H10B	0.4157	0.8096	1.2778	0.023*	
C11	0.60476 (11)	0.90032 (8)	1.16960 (7)	0.01771 (15)	
H11A	0.6727	0.8897	1.0966	0.021*	
C12	0.49358 (13)	1.02704 (9)	1.15505 (8)	0.02153 (16)	
H12A	0.5671	1.0909	1.1330	0.032*	
H12B	0.4201	1.0371	1.2246	0.032*	
H12C	0.4236	1.0329	1.0984	0.032*	
C15	0.52183 (11)	0.75344 (8)	0.65702 (7)	0.01516 (14)	
H15A	0.5176	0.8354	0.6196	0.018*	
C16	0.68701 (10)	0.67289 (8)	0.65650 (7)	0.01452 (13)	
C17	0.70399 (11)	0.55401 (8)	0.72377 (7)	0.01873 (15)	
H17A	0.6074	0.5249	0.7704	0.022*	
C18	0.86260 (12)	0.48024 (9)	0.72112 (8)	0.02101 (16)	
H18A	0.8731	0.4024	0.7669	0.025*	
C19	1.00817 (11)	0.52267 (8)	0.64930 (7)	0.01772 (15)	
C20	0.99354 (11)	0.63973 (8)	0.58088 (7)	0.01699 (14)	
H20A	1.0895	0.6678	0.5327	0.020*	
C21	0.83293 (11)	0.71389 (8)	0.58586 (7)	0.01655 (14)	
H21A	0.8229	0.7924	0.5411	0.020*	
C22	1.30675 (12)	0.47650 (9)	0.57506 (8)	0.02174 (16)	
H22A	1.3986	0.4081	0.5787	0.033*	
H22B	1.3432	0.5464	0.5936	0.033*	
H22C	1.2787	0.4981	0.5007	0.033*	
H1N1	−0.0570 (19)	0.9791 (14)	0.6181 (12)	0.032 (4)*	

### Atomic displacement parameters (Å^2^)

**Table:**

	*U*^11^	*U*^22^	*U*^33^	*U*^12^	*U*^13^	*U*^23^
S1	0.01509 (9)	0.02322 (11)	0.01447 (9)	0.00021 (7)	−0.00279 (6)	−0.00051 (7)
O1	0.0149 (3)	0.0231 (3)	0.0274 (3)	0.0035 (2)	−0.0003 (2)	0.0004 (3)
N1	0.0139 (3)	0.0221 (3)	0.0159 (3)	0.0014 (3)	−0.0040 (2)	−0.0045 (2)
N2	0.0143 (3)	0.0277 (4)	0.0161 (3)	−0.0012 (3)	−0.0027 (2)	−0.0057 (3)
N3	0.0122 (3)	0.0167 (3)	0.0142 (3)	−0.0006 (2)	−0.0029 (2)	−0.0028 (2)
N4	0.0137 (3)	0.0163 (3)	0.0160 (3)	0.0004 (2)	−0.0041 (2)	−0.0028 (2)
C1	0.0137 (3)	0.0164 (3)	0.0148 (3)	−0.0007 (3)	−0.0043 (2)	−0.0037 (3)
C2	0.0133 (3)	0.0229 (4)	0.0148 (3)	−0.0038 (3)	−0.0024 (2)	−0.0041 (3)
C3	0.0142 (5)	0.0172 (6)	0.0143 (4)	−0.0034 (4)	−0.0019 (3)	−0.0028 (4)
C14	0.0181 (4)	0.0339 (6)	0.0210 (4)	−0.0111 (4)	−0.0002 (3)	−0.0054 (4)
C3A	0.018 (6)	0.017 (6)	0.026 (6)	−0.002 (5)	−0.008 (4)	0.002 (5)
C14A	0.044 (8)	0.047 (8)	0.025 (5)	−0.025 (6)	−0.012 (5)	0.003 (5)
C13	0.0234 (4)	0.0274 (5)	0.0288 (4)	−0.0026 (4)	−0.0101 (4)	−0.0098 (4)
C4	0.0156 (3)	0.0199 (4)	0.0137 (3)	−0.0054 (3)	−0.0023 (3)	−0.0007 (3)
C5	0.0148 (3)	0.0208 (4)	0.0198 (4)	−0.0007 (3)	−0.0028 (3)	−0.0029 (3)
C6	0.0184 (4)	0.0192 (4)	0.0192 (3)	−0.0010 (3)	−0.0015 (3)	−0.0056 (3)
C7	0.0182 (3)	0.0152 (3)	0.0134 (3)	−0.0034 (3)	−0.0030 (3)	−0.0012 (2)
C8	0.0152 (3)	0.0154 (3)	0.0163 (3)	−0.0019 (3)	−0.0035 (3)	−0.0020 (3)
C9	0.0173 (3)	0.0169 (3)	0.0155 (3)	−0.0033 (3)	−0.0018 (3)	−0.0038 (3)
C10	0.0255 (4)	0.0182 (4)	0.0164 (3)	−0.0046 (3)	−0.0076 (3)	−0.0019 (3)
C11	0.0176 (4)	0.0198 (4)	0.0170 (3)	−0.0031 (3)	−0.0031 (3)	−0.0057 (3)
C12	0.0224 (4)	0.0187 (4)	0.0234 (4)	−0.0041 (3)	−0.0035 (3)	−0.0028 (3)
C15	0.0154 (3)	0.0146 (3)	0.0162 (3)	−0.0009 (3)	−0.0050 (3)	−0.0032 (3)
C16	0.0137 (3)	0.0147 (3)	0.0153 (3)	−0.0011 (3)	−0.0032 (2)	−0.0027 (2)
C17	0.0154 (3)	0.0185 (4)	0.0188 (3)	−0.0009 (3)	−0.0010 (3)	0.0015 (3)
C18	0.0173 (4)	0.0188 (4)	0.0220 (4)	0.0000 (3)	−0.0008 (3)	0.0031 (3)
C19	0.0148 (3)	0.0176 (4)	0.0194 (3)	0.0000 (3)	−0.0025 (3)	−0.0025 (3)
C20	0.0146 (3)	0.0163 (3)	0.0196 (3)	−0.0032 (3)	−0.0013 (3)	−0.0027 (3)
C21	0.0164 (3)	0.0142 (3)	0.0187 (3)	−0.0027 (3)	−0.0030 (3)	−0.0018 (3)
C22	0.0152 (4)	0.0257 (4)	0.0237 (4)	0.0000 (3)	−0.0010 (3)	−0.0072 (3)

### Geometric parameters (Å, °)

**Table:**

S1—C1	1.6852 (8)	C5—H5A	0.93
O1—C19	1.3597 (11)	C6—C7	1.4009 (12)
O1—C22	1.4300 (11)	C6—H6A	0.93
N1—C1	1.3399 (11)	C7—C8	1.3932 (12)
N1—N2	1.3781 (11)	C7—C10	1.5098 (12)
N1—H1N1	0.872 (15)	C8—C9	1.3935 (11)
N2—C2	1.3033 (12)	C8—H8A	0.93
N3—C1	1.3813 (10)	C9—H9A	0.93
N3—C2	1.3818 (11)	C10—C11	1.5392 (12)
N3—N4	1.3968 (10)	C10—H10A	0.97
N4—C15	1.2885 (11)	C10—H10B	0.97
C2—C3A	1.425 (15)	C11—C12	1.5257 (13)
C2—C3	1.5075 (16)	C11—H11A	0.98
C3—C4	1.5307 (15)	C12—H12A	0.96
C3—C14	1.5360 (17)	C12—H12B	0.96
C3—H3A	0.98	C12—H12C	0.96
C14—H14A	0.96	C15—C16	1.4561 (12)
C14—H14B	0.96	C15—H15A	0.93
C14—H14C	0.96	C16—C21	1.3945 (11)
C3A—C14A	1.47 (2)	C16—C17	1.4068 (12)
C3A—C4	1.492 (14)	C17—C18	1.3793 (12)
C3A—H3AA	0.98	C17—H17A	0.93
C14A—H14D	0.96	C18—C19	1.4038 (12)
C14A—H14E	0.96	C18—H18A	0.93
C14A—H14F	0.96	C19—C20	1.3958 (12)
C13—C11	1.5295 (13)	C20—C21	1.3935 (12)
C13—H13A	0.96	C20—H20A	0.930
C13—H13B	0.96	C21—H21A	0.93
C13—H13C	0.96	C22—H22A	0.96
C4—C9	1.3876 (12)	C22—H22B	0.96
C4—C5	1.3983 (13)	C22—H22C	0.96
C5—C6	1.3968 (12)		
			
C19—O1—C22	117.43 (7)	C8—C7—C10	119.69 (8)
C1—N1—N2	113.71 (7)	C6—C7—C10	122.67 (8)
C1—N1—H1N1	127.2 (10)	C7—C8—C9	121.59 (8)
N2—N1—H1N1	118.4 (10)	C7—C8—H8A	119.2
C2—N2—N1	104.40 (7)	C9—C8—H8A	119.2
C1—N3—C2	108.44 (7)	C4—C9—C8	120.72 (8)
C1—N3—N4	129.86 (7)	C4—C9—H9A	119.6
C2—N3—N4	120.95 (7)	C8—C9—H9A	119.6
C15—N4—N3	115.36 (7)	C7—C10—C11	115.19 (7)
N1—C1—N3	102.87 (7)	C7—C10—H10A	108.5
N1—C1—S1	127.94 (7)	C11—C10—H10A	108.5
N3—C1—S1	129.04 (6)	C7—C10—H10B	108.5
N2—C2—N3	110.38 (7)	C11—C10—H10B	108.5
N2—C2—C3A	111.2 (7)	H10A—C10—H10B	107.5
N3—C2—C3A	137.2 (6)	C12—C11—C13	110.49 (7)
N2—C2—C3	126.82 (9)	C12—C11—C10	112.14 (7)
N3—C2—C3	122.71 (9)	C13—C11—C10	109.89 (7)
C2—C3—C4	111.44 (10)	C12—C11—H11A	108.1
C2—C3—C14	109.65 (11)	C13—C11—H11A	108.1
C4—C3—C14	112.38 (9)	C10—C11—H11A	108.1
C2—C3—H3A	107.7	C11—C12—H12A	109.5
C4—C3—H3A	107.7	C11—C12—H12B	109.5
C14—C3—H3A	107.7	H12A—C12—H12B	109.5
C2—C3A—C14A	103.8 (12)	C11—C12—H12C	109.5
C2—C3A—C4	118.7 (10)	H12A—C12—H12C	109.5
C14A—C3A—C4	105.1 (10)	H12B—C12—H12C	109.5
C2—C3A—H3AA	109.6	N4—C15—C16	119.97 (7)
C14A—C3A—H3AA	109.6	N4—C15—H15A	120.0
C4—C3A—H3AA	109.6	C16—C15—H15A	120.0
C3A—C14A—H14D	109.5	C21—C16—C17	118.75 (8)
C3A—C14A—H14E	109.5	C21—C16—C15	119.08 (7)
H14D—C14A—H14E	109.5	C17—C16—C15	122.16 (7)
C3A—C14A—H14F	109.5	C18—C17—C16	120.57 (8)
H14D—C14A—H14F	109.5	C18—C17—H17A	119.7
H14E—C14A—H14F	109.5	C16—C17—H17A	119.7
C11—C13—H13A	109.5	C17—C18—C19	119.99 (8)
C11—C13—H13B	109.5	C17—C18—H18A	120.0
H13A—C13—H13B	109.5	C19—C18—H18A	120.0
C11—C13—H13C	109.5	O1—C19—C20	124.62 (8)
H13A—C13—H13C	109.5	O1—C19—C18	115.08 (8)
H13B—C13—H13C	109.5	C20—C19—C18	120.30 (8)
C9—C4—C5	118.33 (8)	C21—C20—C19	118.99 (8)
C9—C4—C3A	137.9 (7)	C21—C20—H20A	120.5
C5—C4—C3A	103.8 (7)	C19—C20—H20A	120.5
C9—C4—C3	119.03 (9)	C20—C21—C16	121.39 (8)
C5—C4—C3	122.60 (9)	C20—C21—H21A	119.3
C6—C5—C4	120.85 (8)	C16—C21—H21A	119.3
C6—C5—H5A	119.6	O1—C22—H22A	109.5
C4—C5—H5A	119.6	O1—C22—H22B	109.5
C5—C6—C7	120.84 (8)	H22A—C22—H22B	109.5
C5—C6—H6A	119.6	O1—C22—H22C	109.5
C7—C6—H6A	119.6	H22A—C22—H22C	109.5
C8—C7—C6	117.63 (8)	H22B—C22—H22C	109.5
			
C1—N1—N2—C2	1.66 (10)	C14—C3—C4—C9	129.28 (11)
C1—N3—N4—C15	41.04 (12)	C2—C3—C4—C5	75.33 (12)
C2—N3—N4—C15	−150.12 (8)	C14—C3—C4—C5	−48.21 (15)
N2—N1—C1—N3	−3.89 (9)	C2—C3—C4—C3A	62.7 (18)
N2—N1—C1—S1	171.98 (6)	C14—C3—C4—C3A	−60.8 (18)
C2—N3—C1—N1	4.54 (9)	C9—C4—C5—C6	−1.79 (13)
N4—N3—C1—N1	174.46 (8)	C3A—C4—C5—C6	179.9 (6)
C2—N3—C1—S1	−171.28 (7)	C3—C4—C5—C6	175.71 (9)
N4—N3—C1—S1	−1.35 (13)	C4—C5—C6—C7	1.00 (13)
N1—N2—C2—N3	1.41 (9)	C5—C6—C7—C8	0.72 (12)
N1—N2—C2—C3A	171.1 (7)	C5—C6—C7—C10	−178.53 (8)
N1—N2—C2—C3	−175.19 (9)	C6—C7—C8—C9	−1.67 (12)
C1—N3—C2—N2	−3.89 (10)	C10—C7—C8—C9	177.61 (8)
N4—N3—C2—N2	−174.88 (7)	C5—C4—C9—C8	0.86 (12)
C1—N3—C2—C3A	−169.6 (10)	C3A—C4—C9—C8	178.3 (8)
N4—N3—C2—C3A	19.4 (10)	C3—C4—C9—C8	−176.73 (9)
C1—N3—C2—C3	172.88 (9)	C7—C8—C9—C4	0.89 (12)
N4—N3—C2—C3	1.89 (13)	C8—C7—C10—C11	96.95 (10)
N2—C2—C3—C4	−107.03 (11)	C6—C7—C10—C11	−83.81 (11)
N3—C2—C3—C4	76.76 (13)	C7—C10—C11—C12	68.35 (10)
C3A—C2—C3—C4	−65.9 (18)	C7—C10—C11—C13	−168.35 (8)
N2—C2—C3—C14	18.05 (15)	N3—N4—C15—C16	−179.10 (7)
N3—C2—C3—C14	−158.17 (9)	N4—C15—C16—C21	170.31 (8)
C3A—C2—C3—C14	59.2 (18)	N4—C15—C16—C17	−8.71 (12)
N2—C2—C3A—C14A	113.0 (10)	C21—C16—C17—C18	0.99 (13)
N3—C2—C3A—C14A	−81.3 (12)	C15—C16—C17—C18	−179.99 (8)
C3—C2—C3A—C14A	−32.6 (14)	C16—C17—C18—C19	−1.21 (14)
N2—C2—C3A—C4	−130.8 (10)	C22—O1—C19—C20	−4.71 (13)
N3—C2—C3A—C4	34.9 (18)	C22—O1—C19—C18	175.19 (8)
C3—C2—C3A—C4	83.6 (19)	C17—C18—C19—O1	−179.50 (8)
C2—C3A—C4—C9	−73.2 (14)	C17—C18—C19—C20	0.41 (14)
C14A—C3A—C4—C9	42.3 (15)	O1—C19—C20—C21	−179.51 (8)
C2—C3A—C4—C5	104.5 (11)	C18—C19—C20—C21	0.59 (13)
C14A—C3A—C4—C5	−140.0 (10)	C19—C20—C21—C16	−0.81 (13)
C2—C3A—C4—C3	−86 (2)	C17—C16—C21—C20	0.03 (13)
C14A—C3A—C4—C3	29.1 (12)	C15—C16—C21—C20	−179.02 (8)
C2—C3—C4—C9	−107.18 (11)		

### Hydrogen-bond geometry (Å, °)

**Table:**

*D*—H···*A*	*D*—H	H···*A*	*D*···*A*	*D*—H···*A*
N1—H1N1···S1^i^	0.87 (2)	2.39 (2)	3.2482 (8)	168 (1)
C15—H15A···S1	0.93	2.66	3.1947 (9)	117
C13—H13A···Cg1^ii^	0.96	2.77	3.5416 (12)	138
C12—H12A···Cg2^ii^	0.96	2.73	3.5417 (11)	142
C18—H18A···Cg2^iii^	0.93	2.80	3.5903 (10)	144
C22—H22C···Cg3^iv^	0.96	2.78	3.5783 (10)	142
C14A—H14D···Cg3^v^	0.96	2.88	3.769 (13)	155

Symmetry codes: (i) −*x*, −*y*+2, −*z*+1; (ii) −*x*+1, −*y*+2, −*z*+2; (iii) −*x*+1, −*y*+1, −*z*+2; (iv) −*x*+2, −*y*+1, −*z*+1; (v) *x*−1, *y*, *z*.

## References

[bb1] Allen, F. H., Kennard, O., Watson, D. G., Brammer, L., Orpen, A. G. & Taylor, R. (1987). *J. Chem. Soc. Perkin Trans. 2*, pp. S1–19.

[bb2] Amir, M. & Kumar, S. (2007). *Acta Pharm.* **57**, 31–45. 10.2478/v10007-007-0003-y 19839405

[bb3] Bernstein, J., Davis, R. E., Shimoni, L. & Chang, N.-L. (1995). *Angew. Chem. Int. Ed. Engl.* **34**, 1555–1573.

[bb4] Bruker (2005). *APEX2*, *SAINT* and *SADABS* Bruker AXS Inc., Madison, Wisconsin, USA.

[bb5] Clemons, M., Coleman, R. E. & Verma, S. (2004). *Cancer Treat. Rev.* **30**, 325–332. 10.1016/j.ctrv.2004.03.004 15145507

[bb6] Cosier, J. & Glazer, A. M. (1986). *J. Appl. Cryst.* **19**, 105–107.

[bb7] Demirbas, N. & Ugurluoglu, R. (2004). *Turk. J. Chem.* **28**, 679–690.

[bb8] Demirbas, N., Ugurluoglu, R. & Demirbasx, A. (2002). *Bioorg. Med. Chem.* **10**, 3717–3723. 10.1016/s0968-0896(02)00420-0 12413828

[bb9] Johnston, G. A. R. (2002). *Curr. Top. Med. Chem.* **2**, 903–913. 10.2174/1568026023393453 12171579

[bb10] Sheldrick, G. M. (2008). *Acta Cryst.* A**64**, 112–122. 10.1107/S0108767307043930 18156677

[bb11] Shujuan, S., Hongxiang, L., Gao, Y., Fan, P., Ma, B., Ge, W. & Wang, X. (2004). *J. Pharm. Biomed. Anal.* **34**, 1117–1124. 10.1016/j.jpba.2003.11.013 15019046

[bb12] Spek, A. L. (2009). *Acta Cryst.* D**65**, 148–155. 10.1107/S090744490804362X PMC2631630 19171970

